# A Model for Selection of Eyespots on Butterfly Wings

**DOI:** 10.1371/journal.pone.0141434

**Published:** 2015-11-04

**Authors:** Toshio Sekimura, Chandrasekhar Venkataraman, Anotida Madzvamuse

**Affiliations:** 1 Department of Biological Chemistry, Graduate School of Bioscience and Biotechnology, Chubu University, Kasugai, Aichi 487–8501, Japan; 2 School of Mathematics and Statistics, University of St Andrews, Fife, KY16 9SS, United Kingdom; 3 Department of Mathematics, University of Sussex, Falmer, BN1 9QH, United Kingdom; Universitat Pompeu Fabra, SPAIN

## Abstract

**Unsolved Problem:**

The development of eyespots on the wing surface of butterflies of the family Nympalidae is one of the most studied examples of biological pattern formation.However, little is known about the mechanism that determines the number and precise locations of eyespots on the wing. Eyespots develop around signaling centers, called foci, that are located equidistant from wing veins along the midline of a wing cell (an area bounded by veins). A fundamental question that remains unsolved is, why a certain wing cell develops an eyespot, while other wing cells do not.

**Key Idea and Model:**

We illustrate that the key to understanding focus point selection may be in the venation system of the wing disc. Our main hypothesis is that changes in morphogen concentration along the proximal boundary veins of wing cells govern focus point selection. Based on previous studies, we focus on a spatially two-dimensional reaction-diffusion system model posed in the interior of each wing cell that describes the formation of focus points. Using finite element based numerical simulations, we demonstrate that variation in the proximal boundary condition is sufficient to robustly select whether an eyespot focus point forms in otherwise identical wing cells. We also illustrate that this behavior is robust to small perturbations in the parameters and geometry and moderate levels of noise. Hence, we suggest that an anterior-posterior pattern of morphogen concentration along the proximal vein may be the main determinant of the distribution of focus points on the wing surface. In order to complete our model, we propose a two stage reaction-diffusion system model, in which an one-dimensional surface reaction-diffusion system, posed on the proximal vein, generates the morphogen concentrations that act as non-homogeneous Dirichlet (i.e., fixed) boundary conditions for the two-dimensional reaction-diffusion model posed in the wing cells. The two-stage model appears capable of generating focus point distributions observed in nature.

**Result:**

We therefore conclude that changes in the proximal boundary conditions are sufficient to explain the empirically observed distribution of eyespot focus points on the entire wing surface. The model predicts, subject to experimental verification, that the source strength of the activator at the proximal boundary should be lower in wing cells in which focus points form than in those that lack focus points. The model suggests that the number and locations of eyespot foci on the wing disc could be largely controlled by two kinds of gradients along two different directions, that is, the first one is the gradient in spatially varying parameters such as the reaction rate along the anterior-posterior direction on the proximal boundary of the wing cells, and the second one is the gradient in source values of the activator along the veins in the proximal-distal direction of the wing cell.

## Introduction

**Fig 1:**
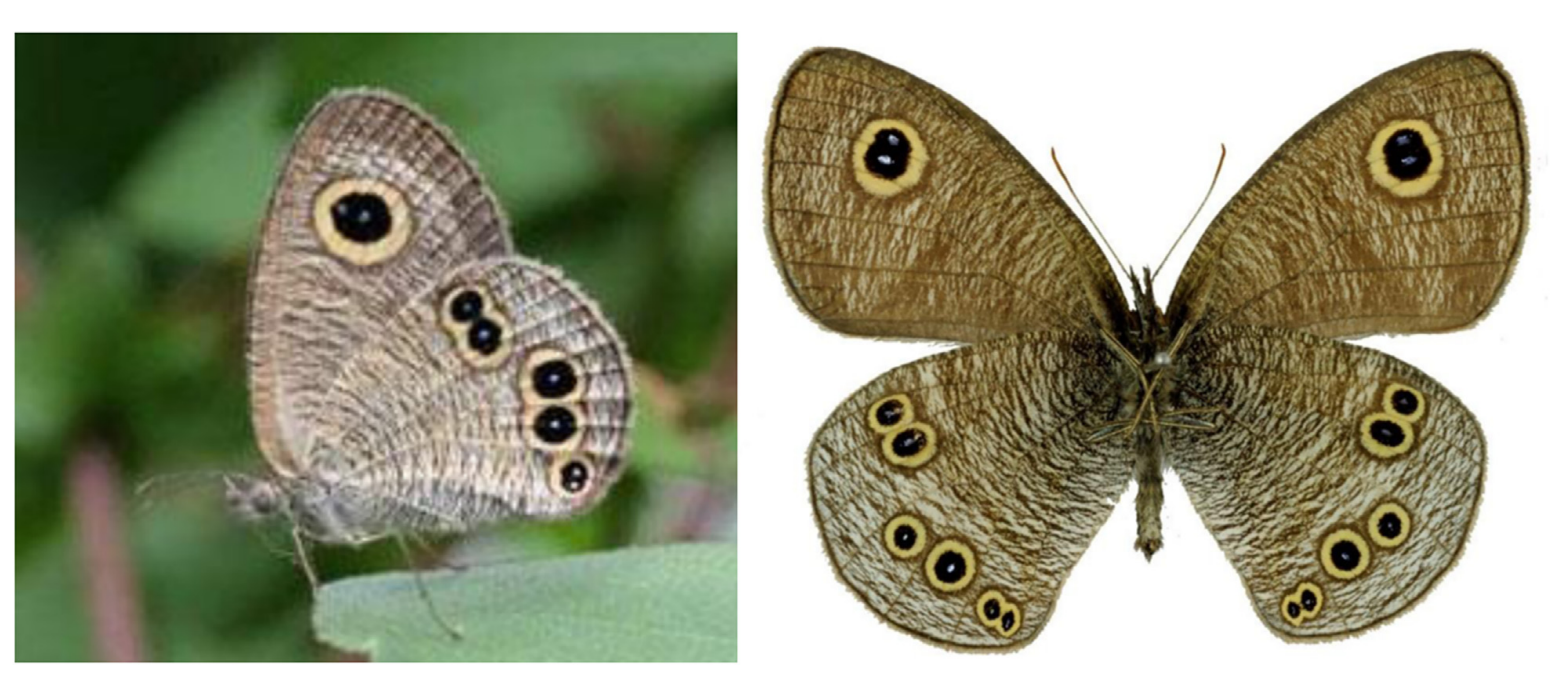
Ventral eyespot patterns of the butterfly *Ypthima arugus* (Nymphalidae, Satyrinae) at rest (left), and the extended adult specimen (right). The right-hand side photo: courtesy of Mr.Toru Tokiwa.

Butterfly wing color patterns are among the most spectacular and remarkable examples of patterning in biology. For more than a century, they have attracted much attention from experimentalists and theoreticians alike. One of the most studied color patterns on butterfly wings is the eyespot (Fig 1) that may play a central role in interactions with predators. The formation of eyespots has been the subject of studies in molecular and developmental genetics (e.g.,[1,2,3]), evolution, physiology (e.g., [4, 5]), ecology (e.g., [6, 7, 8]), and theoretical biology (e.g., [9, 10, 11]). These studies, however, have focused on the formation mechanism of a single eyespot located at a specific position on the wing surface. Several species of butterflies, however, have many eyespots on their wing surface. The number, size, shape, pigmentation and precise position of these eyespots are extremely diverse and are typically species-specific. In order to fully understand the evolution and diversity of eyespot patterns, it is necessary to analyze the mechanism that governs the formation of these different pattern elements.

In this paper, we focus on the mechanism underlying the determination of the number and locations of eyespots on the wing surface. Each eyespot develops around a focus, a small group of cells that sends out a morphogenetic signal that determines the synthesis of circular patterns of pigments in their surroundings. Our paper is concerned with the mechanism that places these foci in various locations on the wing surface. This fundamental process constitutes the first of three developmental steps of eyespot formation (see Section 2 for details). We do not consider the mechanism behind the determination of the size, shape and pigment patterns around the foci, which occurs later in the developmental stages. The number and locations of foci would have undergone considerable evolution during the diversification of the butterflies. Our objective in this article is to propose a model that determines the global distribution of foci in the overall venation system of the wing.

## Background and a Mechanism for Selection of Focus Points

### 2.1 General features of eyespot formation

The formation of wing color patterns including eyespot patterns is a spatially two-dimensional phenomenon that takes place in the single layer of cells that makes up each surface of the wing [e.g., 12]. The butterfly wing begins its development as a wing imaginal disc in the larva. The wing imaginal disc is transparent and colorless throughout the larval and early pupal stages of development. Antibody and mRNA fluorescence techniques for the several developmental genes have revealed existence of a developmental pre-pattern on the wing disc, which predicts the color pattern of the adult butterfly wing (e.g., [3]).

For the specific case of eyespot formation, the formation mechanism is thought to consist of the following three developmental stages (e.g., [13, 14])

- The first stage is the determination of the location of the signaling center, i.e., “the eyespot focal cells”, from which some signaling chemicals, or morphogens originate.
- The second stage is the spreading out of morphogens into the surroundings of the focus cells through diffusion and activation of corresponding genes (e.g., *Dll*, *engrailed*), which establish a pattern of concentric rings of gene expression that constitutes the pre-pattern for pigment synthesis.
- The third stage is the activation of the pigmentation genes (e.g., *DDC*, *GTP-CH1*, *cinebar*) that cause the synthesis of species-specific pigments as a set of concentric colored rings we recognize as an eyespot. The focus cells are typically pigmented white and form the “pupil” of the eyespot on the adult wing.

The target problem in this paper pertains to the first developmental stage described above: the formation and positioning of the foci. We will not consider the growth of the wing disc as part of the modeling as this is assumed to occur on a longer timescale influencing only the second and the third stages.

### 2.2 A mechanism for selection of eyespot focus points in the wing disc

**Fig 2:**
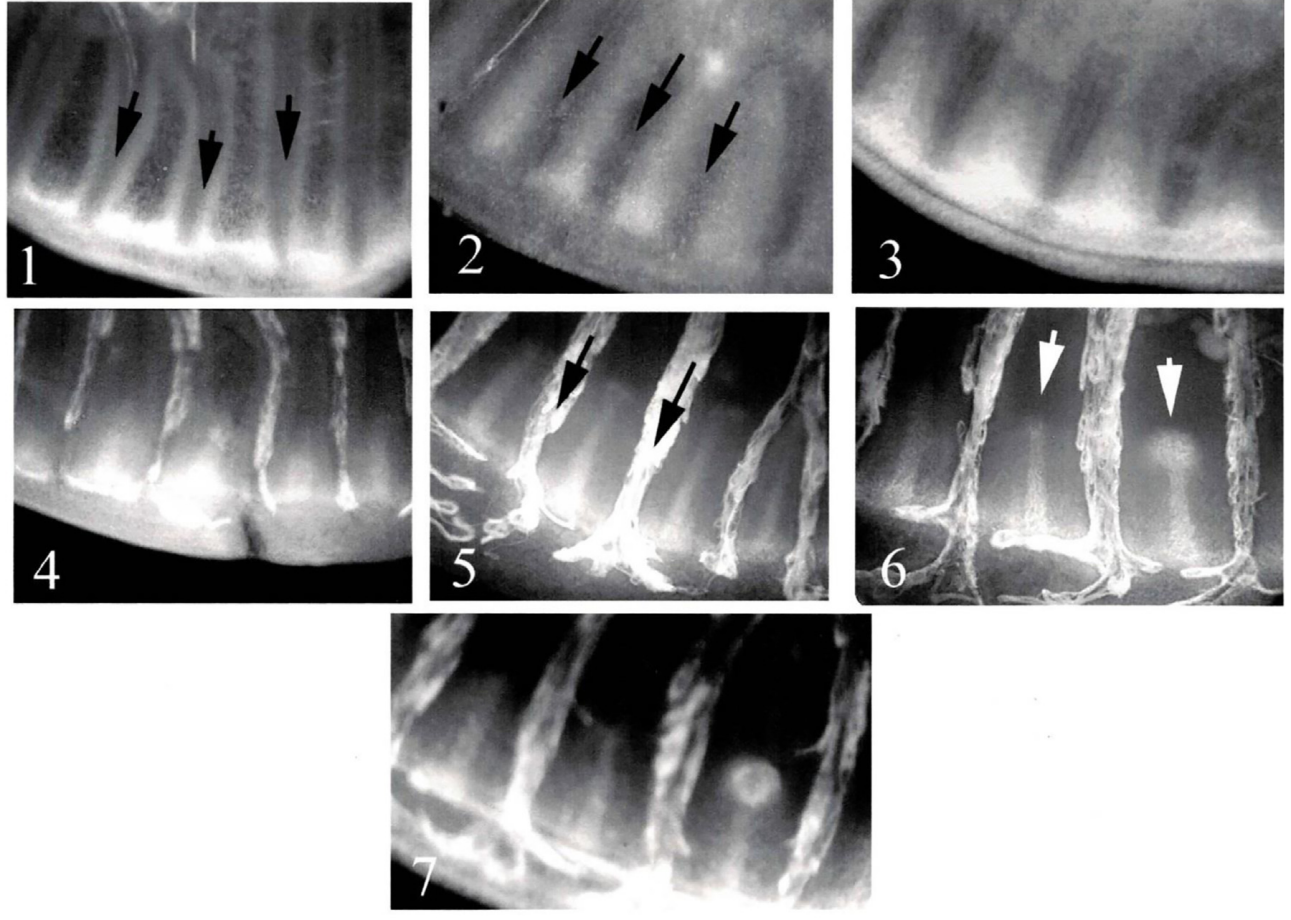
Development of eyespot focus points in the wing disc of *Junonia coenia* (Nymphalidae, Nymphalinae). Numbers 1~7 in the photos show a time course of the *Notch* expression pattern during the focus point development. The expression pattern by antibody staining were visualized on a fluorescent light microscope and digitally photographed. Black arrows in photo numbers 1, 2, and 5 indicate pre-veins, which finally evolve to become veins of the adult butterfly wing. White arrows in photo 6 show two peaks of *N*-related chemicals along the centerline of each wing cell, the right-hand one of which evolves into a focus point afterwards (in photo number 7) while no focus point remains on the left-hand wing cell. Photos: courtesy of Prof. Fred Nijhout of Duke University. For more details on the adult forewing of *J*.*coenia* butterfly, see Fig 7 in Section 3.3.

**Fig 3:**
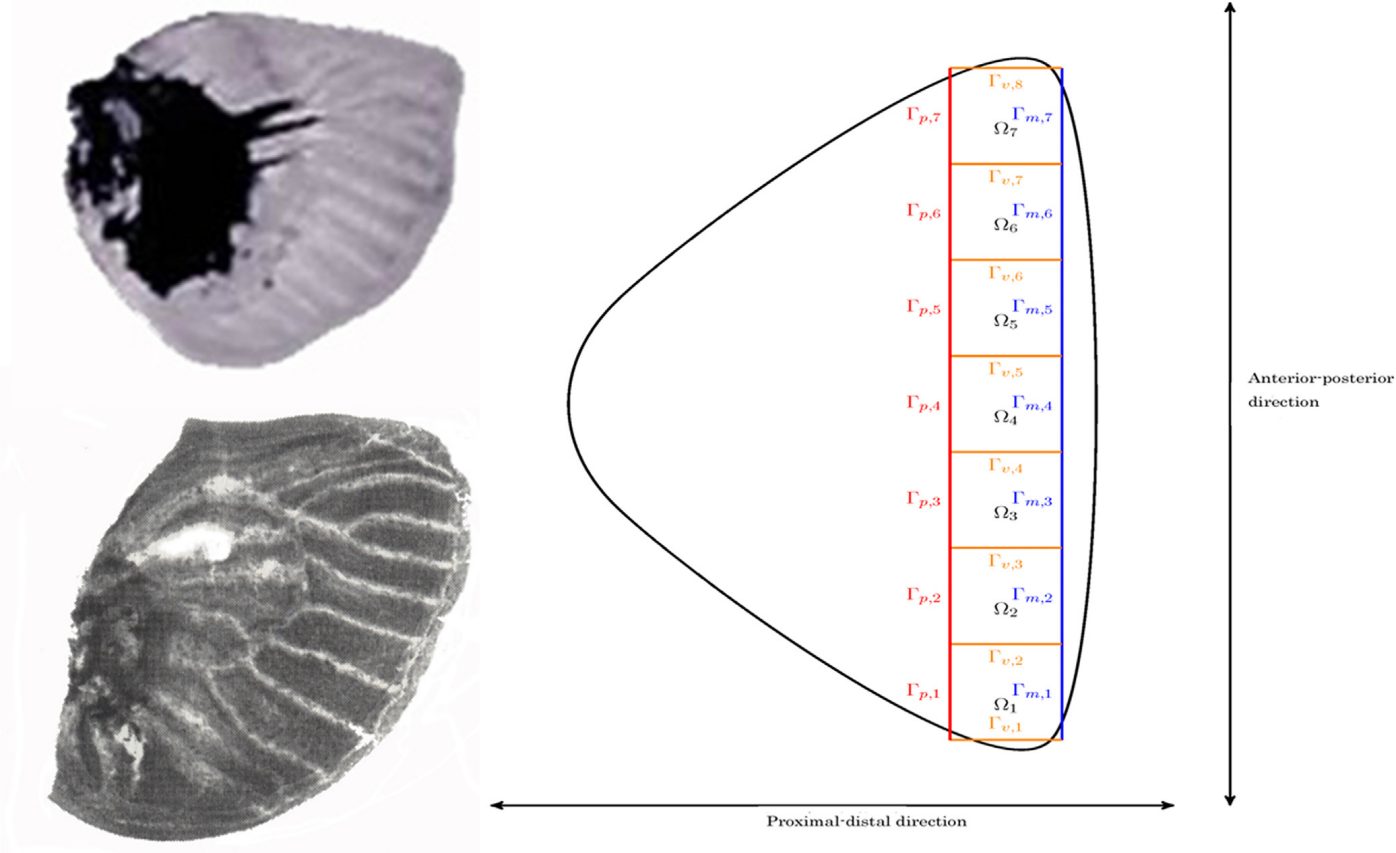
A wing disc in the larval stage (up left) and its venation system (down left) of *Papilio polyxenes* (Papilionidae). Both photos: courtesy of Prof. Fred Nijhout of Duke University. (Right) Hypothetical wing disc and its venation system with rectangular approximation to wing cells in which eyespot focus point selection occurs.

Although there is experimental data on the development of eyespot foci, little is known about the mechanism that determines the number and locations of focus points in the entire wing disc. As seen in Fig 2, only certain wing cells develop eyespot foci, while other wing cells do not develop any foci. The proposal of a mechanism that explains whether or not an eyespot focus forms in a given wing cell is one of the main objectives of the current study. We assume that the key determinant of focus point selection is in the overall venation system of the wing disc. Following Nijhout [9], we assume that veins act as sources of one of the two diffusing reactants. To investigate the selection mechanism, we assume a hypothetical venation system, where wing cells of the wing disc are rectangular (see Fig 3), although for completeness we also illustrate that our results are robust to perturbations from this rectangular geometry by considering wing cells with curved boundaries and varying width in Section 3.2. Under these assumptions, we investigate whether the nature of the proximal boundary condition can determine focus point selection, i.e., the number and locations of focus points in the entire wing disc.

## Proximal Boundary Conditions as a Mechanism for Focus Point Selection

### 3.1 Mathematical description of the model

For the sake of simplicity, we approximate the wing cells by rectangular domains and assume that all wing cells are of the same size (see Fig 3) and are independent [4]. Our results appear insensitive to small perturbations to this geometry. The only difference between wing cells is assumed to be in the source value of the activator at the proximal veins. We propose variable boundary conditions on the proximal boundary in the anterior-posterior direction of the wing disc. We assume that the entire wing disc comprises seven wing cells on which eyespot foci patterns can form. Following Nijhout [9], in the interior of each wing cell, we employ the activator-inhibitor reaction-diffusion model of Gierer-Meinhardt (G-M) [15] that describes focus point formation. Therefore, our model consists of several sets of coupled G-M models; each set is posed in a single rectangular wing cell. We remark that under this framework, the focus point formation occurs independently in each rectangular wing cell.

Let *n*_*seq*_ denote the number of wing cells, typically seven. For the *i*–th (*i* = 1,…,*n*_*seq*_) wing cell, the boundary conditions for the activator concentration (*a*_1_) are Dirichlet (fixed) on the proximal boundary Γ_*p*,*i*_ and the wing veins Γ_*v*,*i*_, Γ_*v*,*i*+1_, and Neumann (zero flux) boundary conditions on the wing margin Γ_*m*,*i*_ (*i* = 1,…,*n*_*seq*_) (see Fig 3). The boundary conditions for the inhibitor concentration (*a*_2_) are zero flux on all four boundaries of each rectangular wing cell. The Dirichlet boundary condition on each vein Γ_*v*,*i*_ is the same for each vein. The initial conditions are taken to be the spatially homogeneous positive steady state solutions of the G-M equation. Thus our model for selection of focus points consists of *n*_*seq*_ independent G-M equations. Let us denote by Ω_*i*_ the *i*-th wing cell with boundaries, Γ_*m*,*i*_ (wing margin), Γ_*v*,*i*_, Γ_*v*,*i*+1_ (veins) and Γ_*p*,*i*_ (proximal boundary). The model system equations in dimensionless form may be stated as follows: For *i* (= 1,…,*n*_*seq*_), we find $\vec{a}(\vec{x},t)={\left(a_{1}(\vec{x},t),\;a_{2}(\vec{x},t)\right)}^{T}$, $\vec{x}\in \Omega_{i}$, such that

$$\left\{ \begin{array}{l} \partial_{t}\vec{a}-\vec{D}\Delta \vec{a}=\vec{f}(\vec{a})\;in\;\Omega_{i},\\ a_{1}(\vec{x},t)=u(\vec{x})\;on\;\Gamma_{p,i},\\ a_{1}(\vec{x},t)=\tilde{a}\;(source\;value)\;on\;\Gamma_{v,i}\cup \Gamma_{v,i+1},\\ \nabla a_{1}\cdot v=0\;(zero\;flux)\;on\;\Gamma_{m,i},\\ \nabla a_{2}\cdot v=0\;(zero\;flux)\;on\;\Gamma_{m,i}\cup \Gamma_{v,i}\cup \Gamma_{v,i+1}\cup \Gamma_{p,i},\\ \vec{a}(\vec{x},0)={\vec{a}}^{ss}\;in\;\Omega_{i}, \end{array}\right.$$ (3.1)

where the reaction function $\vec{f}(\vec{a})$ is given by

$$\left\{ \begin{array}{l} f_{1}(\vec{a})=\alpha \left(\kappa_{1}\frac{a_{1}{}^{2}}{a_{1}}-\kappa_{2}a_{1}\right)\;,\\ f_{2}(\vec{a})=\alpha \left(\kappa_{1}a_{1}{}^{2}-\kappa_{3}a_{2}\right)\;, \end{array}\right.$$ (3.2)

with *α*, *κ*_1_, *κ*_2_, *κ*_3_ > 0. This choice of reaction kinetics implies the existence of a positive steady state ${\vec{a}}^{ss}$ of the ordinary differential equation (ODE) system and this is given by ${\left(\frac{\kappa_{3}}{\kappa_{2}},\frac{\kappa_{1}\kappa_{3}}{\kappa_{2}{}^{2}}\right)}^{T}$.

Other than the prescribed proximal boundary condition $u(\vec{x})$ in Eq (3.1), each of the wing cells is assumed to be identical with the same source terms from the wing veins, diffusion coefficients and reactions. The boundary conditions at the veins are taken to be constant at twice the steady state of the activator, i.e., $\tilde{a}=2\;a_{1}{}^{ss}$ (following [9]).

### 3.2 Simulation results of the model with prescribed boundary conditions

We now present numerical simulations illustrating that the proximal boundary condition can act as a determinant of whether or not a focus point forms in a given wing cell. We use the finite element method, derived and analyzed in Lakkis et al. [16], for all the simulations approximating the equations on meshes with 33025 degrees of freedom and using a time-step of 10^−3^. We take the parameter values for the reaction kinetics and diffusion coefficients to be those given in Table 1. The majority of the results we report on remain qualitatively unchanged with small changes in the parameter values (10%). We consistently show only snapshots of the numerical solution of the activator concentration corresponding to Eq (3.1). The inhibitor concentration profile is in-phase and hence its snapshots are omitted.

**Table 1:** Parameter values used for all the simulations of Eq (3.1).

*D*_1_	*D*_2_	*α*	*κ*_1_	*κ*_2_	*κ*_3_
3.1×10^−3^	3×10^−2^	20	3×10^−2^	3×10^−2^	1.25×10^−2^

We start by considering prescribed boundary conditions on the proximal boundary, i.e., the function $u(\vec{x})$ in Eq (3.1) is a given function. We consider the following three cases for the quadratic proximal boundary condition.

#### 3.2.1 Constant boundary condition

**Fig 4:**
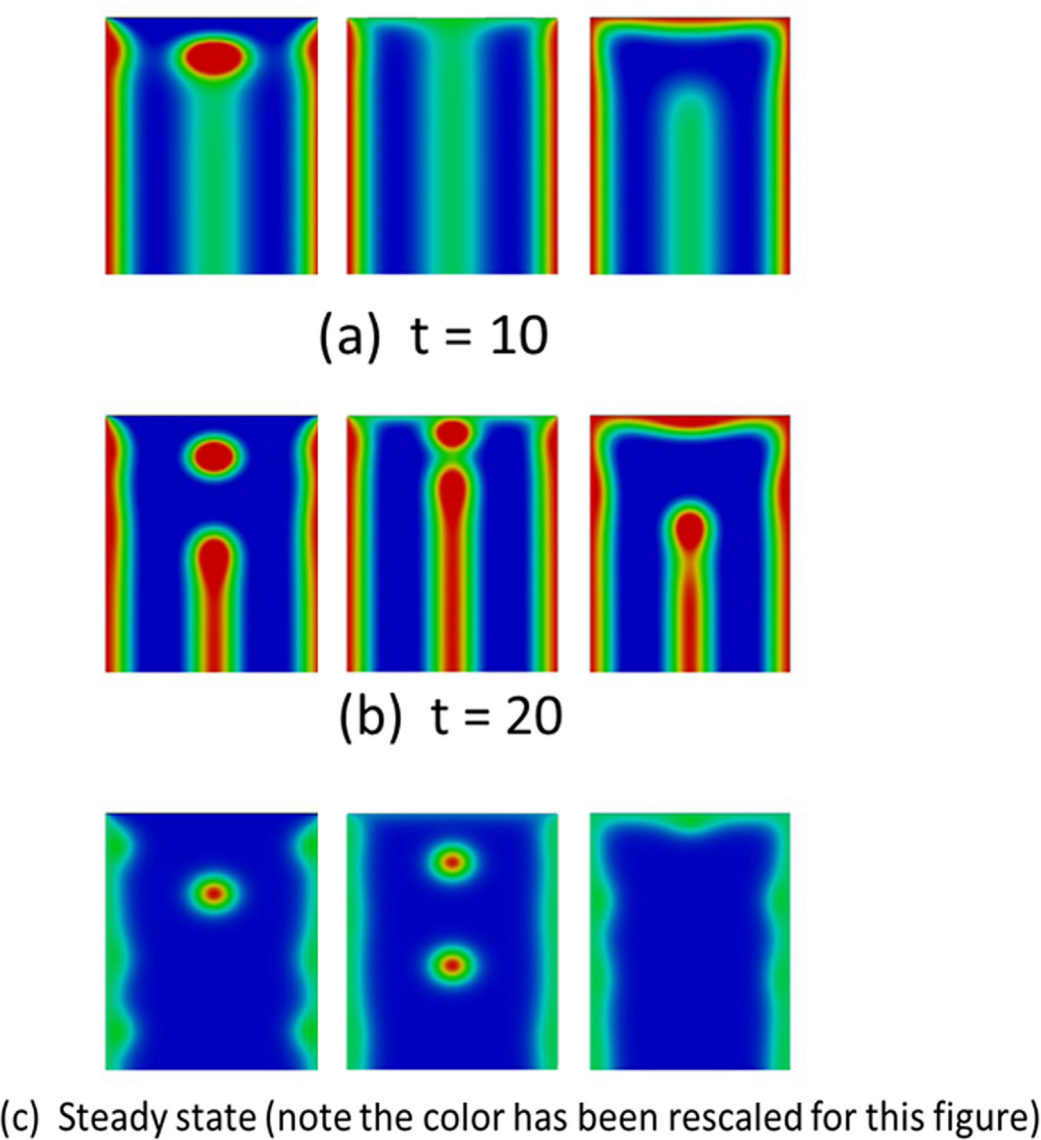
Proximal boundary conditions may govern eyespot focus point determination. The figure shows snapshots of the activator concentration corresponding to the solution of Eq (3.1). The boundary conditions on the proximal boundary (top) of the rectangular cell for the activator are of the form *k*_*p*_ *a*_1_^*ss*^ where *k*_*p*_ = 0, 1 and 2 (reading from left to right in each row) and *a*_1_^*ss*^ is the (activator) steady state value. The veins (left and right boundaries of each wing cell) have Dirichlet (fixed) boundary conditions for the activator with constant values at twice the steady state. Initially in all the wing cells a vertical stripe of high activator concentration is generated originating from the zero-flux distal boundary (bottom). In the wing cells with lowest activator values at the proximal boundary (left hand), a spot forms and this spot eventually moves towards the center of the cell (see also Section 3.3). In the wing cells with medium activator values at the proximal boundary (middle), we have both the formation of a spot from the receding midline peak and later the insertion of a new spot that originates from the proximal boundary with the steady state consisting of two spots. In the wing cell with highest activator values at the proximal boundary (right hand), the vertical stripe recedes without leaving behind a spot.

We first consider the case that the proximal boundary condition is constant in each wing cell, i.e., it is a piecewise constant discontinuous function over the whole proximal anterior-posterior boundary. In Fig 4, we show simulation results of Eq (3.1) on wing cells with constant proximal boundary condition of the form *k*_*p*_ *a*_1_^*ss*^, where *k*_*p*_ = 0, 1 and 2 (reading from left to right in each row) and *a*_1_^*ss*^ is the (activator) steady state value. Each wing cell is taken to be a rectangular domain of length (proximal-wing margin) three and width (anterior-posterior) two. We observe the formation of activator peaks along the centerline of each wing cell (even those that do not eventually possess focus points) that is a characteristic of Nijhout’s model [17] and is observed in experiments [1, 2]. In wing cells with an activator concentration of less than 2*a*_1_^*ss*^ on the proximal boundary, as the midline peak recedes it leaves behind focus points (two columns on the left in Fig 4), whilst for the cell with activator concentration on the proximal boundary equal to 2*a*_1_^*ss*^, (right hand rectangle) the midline peak completely recedes and no focus point remains. Interestingly for the simulations with proximal boundary condition *a*_1_^*ss*^, an extra focus point is formed which originates at the proximal boundary, migrates to the interior of the wing cell and persists at the steady state. Thus, for this choice of parameter values and domain geometry, the number of focus points at steady state does not depend monotonically on the proximal boundary condition. As each wing cell only differs in terms of the proximal boundary condition, we see that the changes in the proximal boundary condition can act as a determinant of focus point formation. The piecewise constant boundary profiles considered so far are only an approximation and it is likely that the real activator boundary profile may appear as a continuous smooth function.

#### 3.2.2 Concave and convex boundary conditions

We consider the following two additional proximal boundary condition profiles, a concave profile:

$$u(\vec{x})=\left(1-{\text{sin}}^{2}\left(\frac{\pi \vec{x}}{w}\right)\right)2a_{1}{}^{ss}\;,$$ (3.3)

and a convex profile:

$$u(\vec{x})=\left({\text{sin}}^{2}\left(\frac{\pi \vec{x}}{w}\right)\right)2a_{1}{}^{ss},$$ (3.4)

where *w* is the width of the wing cell.

**Fig 5:**
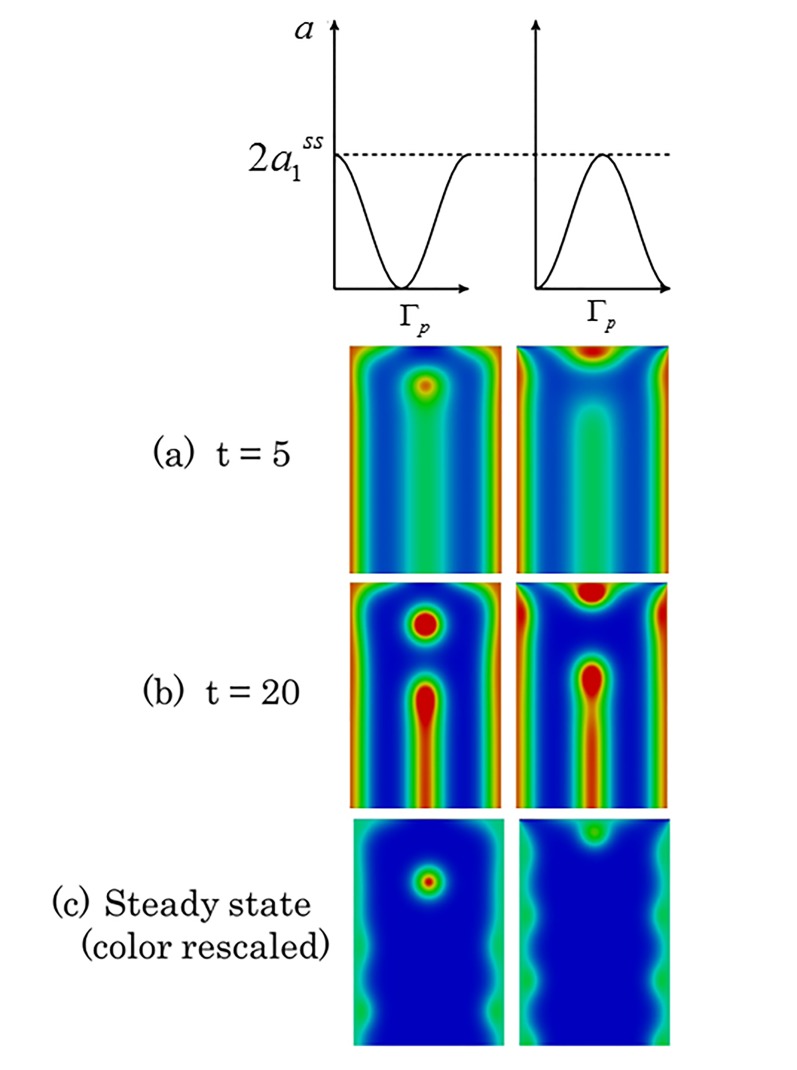
(Top row): Examples of proximal boundary condition: concave (left) and convex (right) profiles. (Bottom row): Numerical simulations on the influence of proximal boundary profile on eyespot focus point determination. The figure shows snapshots of the activator concentration corresponding to the solution of Eq (3.1) on wing cells with proximal boundary conditions. The wing cells are taken to be rectangular of length 3 and width 2. In each subfigure, the left hand plot corresponds to the concave proximal boundary condition and the right hand plot the convex proximal boundary condition (c.f., Fig 5 (Top row)). We observe the formation of a spot in the concave case whilst the midline peak completely recedes leaving behind no spot in the convex case.

Fig 5 shows simulation results of Eq (3.1) on wing cells together with two profiles of the proximal boundary conditions given by Eqs (3.3) and (3.4). As previously, each wing cell is taken to be a rectangular domain of length (proximal-wing margin) three and width (anterior-posterior) two. We once again observe the formation of activator peaks along the centerline of each wing cell and as this midline peak recedes, it leaves behind a spot in the wing cell with the concave boundary condition whilst with the convex boundary condition the peak completely recedes leaving behind no spot. We have performed a number of other simulations (results not shown) with spatially varying (within each wing cell) proximal boundary conditions and we observe analogous behavior to this simulation, namely that the value of the boundary condition in the middle of the proximal boundary of the wing cell is a key in determining whether or not a focus point forms. We further note that by appropriately super imposing together boundary conditions as in Fig 5 together with the piecewise constant boundary conditions used to generate the results of Fig 4, it is possible to generate all possible configurations of focus point distributions (consisting of at most a single focus point in each wing cell) with a boundary profile that is continuous across the whole wing along the anterior-posterior proximal vein.

As a robustness test of sensitivity of the observed behavior to the geometry, we relax the assumption of rectangular geometries and work on a geometry closer to that of the real wing cells shown in Fig 2. We consider a wing cell whose width increases as we move in the proximal-distal direction and we consider curved proximal and wing margin boundaries. The specific geometry for which we present results is defined by the following boundaries: Γ_*v*,1_ is taken to be the line between the points (-0.8, 3) and (-1, 0), Γ_*v*,2_ is taken to be the line between the points (0.83, 3) and (1, 0) and the proximal and wing margin boundaries are taken to be curves given by

$$\Gamma_{p,1}:=\{\vec{x}\in R^{2}|{\left(x_{1}/0.8\right)}^{2}+{\left((x_{2}-3)/0.1\right)}^{2}=1\;with\;x_{2}\leq 3\}$$

and

$$\Gamma_{m,1}:=\{\vec{x}\in R^{2}|{\left(x_{1}\right)}^{2}+{\left((x_{2})/0.1\right)}^{2}=1\;with\;x_{2}\leq 0\}.$$

**Fig 6:**
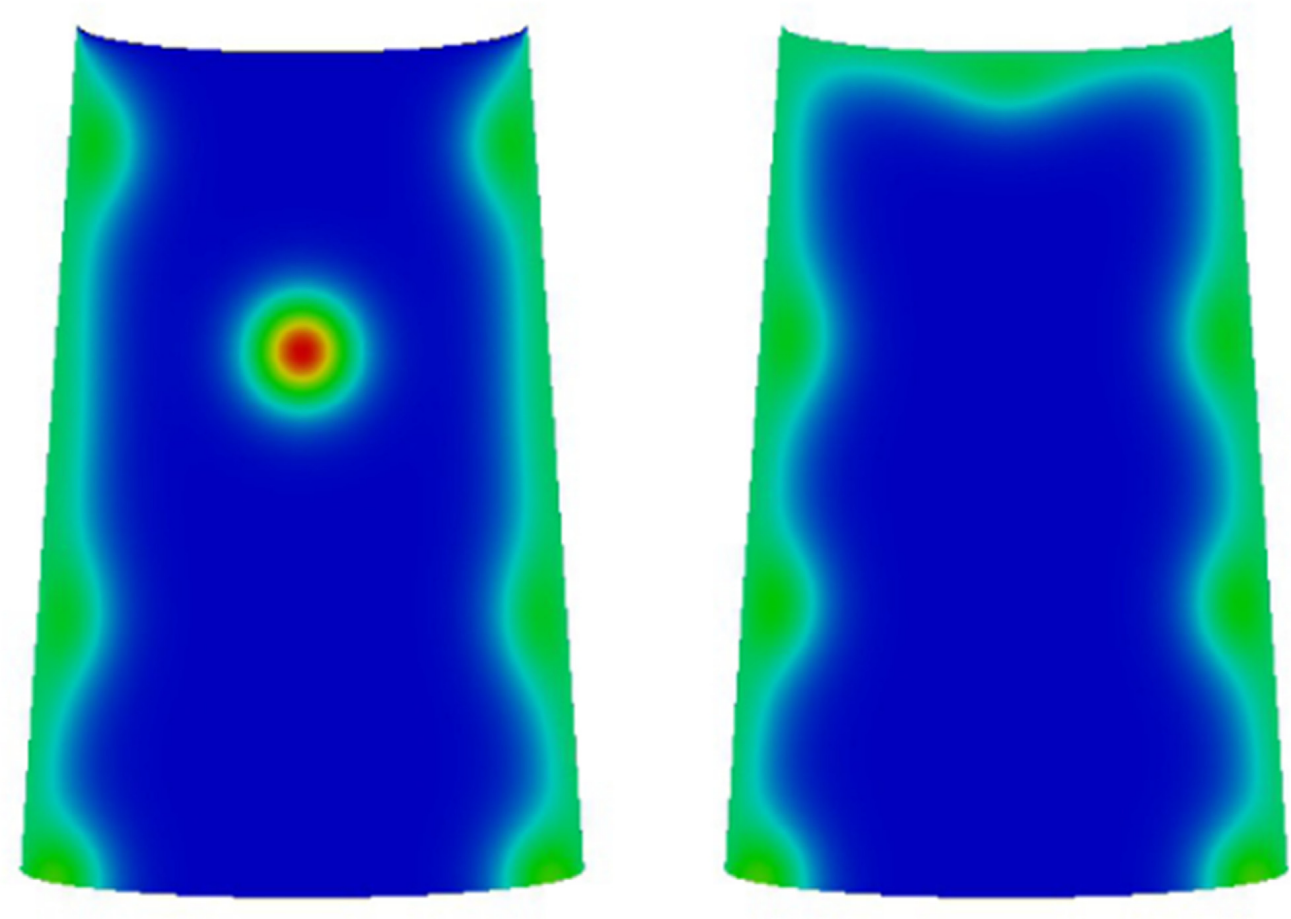
Steady state values of the activator concentration in simulations of Eq (3.1) on a domain of increasing width in the proximal-distal direction (top to bottom) and with curved proximal (top) and wing margin (bottom) boundaries. The left hand figure corresponds to constant boundary conditions equal to zero on the proximal boundary curve. The right hand figure corresponds to proximal boundary conditions equal to twice the activator steady state. The observed behavior is analogous to the rectangular domain case.

Fig 6 shows simulation results of Eq (3.1) on wing cells with curved proximal and wing margin boundaries and increasing width towards the wing margin. We observe analogous behaviors to the rectangular domain case with larger values of the Dirichlet proximal boundary condition inhibiting the formation of a focus point. In the case of values at the steady state for the proximal Dirichlet boundary condition, we note that in contrast to the rectangular domain case only one focus point is observed (results not included in the interests of space), with the steady state profile similar to those of the zero Dirichlet boundary condition (Fig 6 (left)).

Finally, we investigate the dependence of the number of focus points on the aspect ratio of the wing cell (see figures (a)–(h) in S1 Appendix for detailed numerical simulations). Figures (g) and (h) in S1 Appendix show steady states of the simulation of Eq (3.1) on wing cells with constant proximal boundary condition at zero and 2 times the steady state value. The length (proximal-distal) is held fixed at 3 and the width (anterior-posterior) is varied between 1.5 and 3 (i.e., the aspect ratio varies from 2 to 1). We observe a monotonic dependence of the number of focus points on the aspect ratio.

We have also simulated cases (results not shown) where the proximal boundary condition is asymmetric and where the boundary condition along the veins is varied, rather than the fixed Dirichlet conditions presented above. Although it is possible through careful tuning of the parameter values and boundary conditions to generate focus points which are not circular (spots) such as arc shaped foci as well as focus points which are positioned away from the centerline of the wing cell, the predominantly observed behavior is the generation of circular spot shaped foci positioned along the centerline of the wing cell at steady state.

### 3.3 Simulations of variations in focus point patterning observed in nature

We now present some simulation results together with experimental images of real specimens, which illustrate the capability of the model to describe naturally occurring variations in eyespot focus point patterning.

#### 3.3.1 Development of focus points in the wing disc during eyespot determination

**Fig 7:**
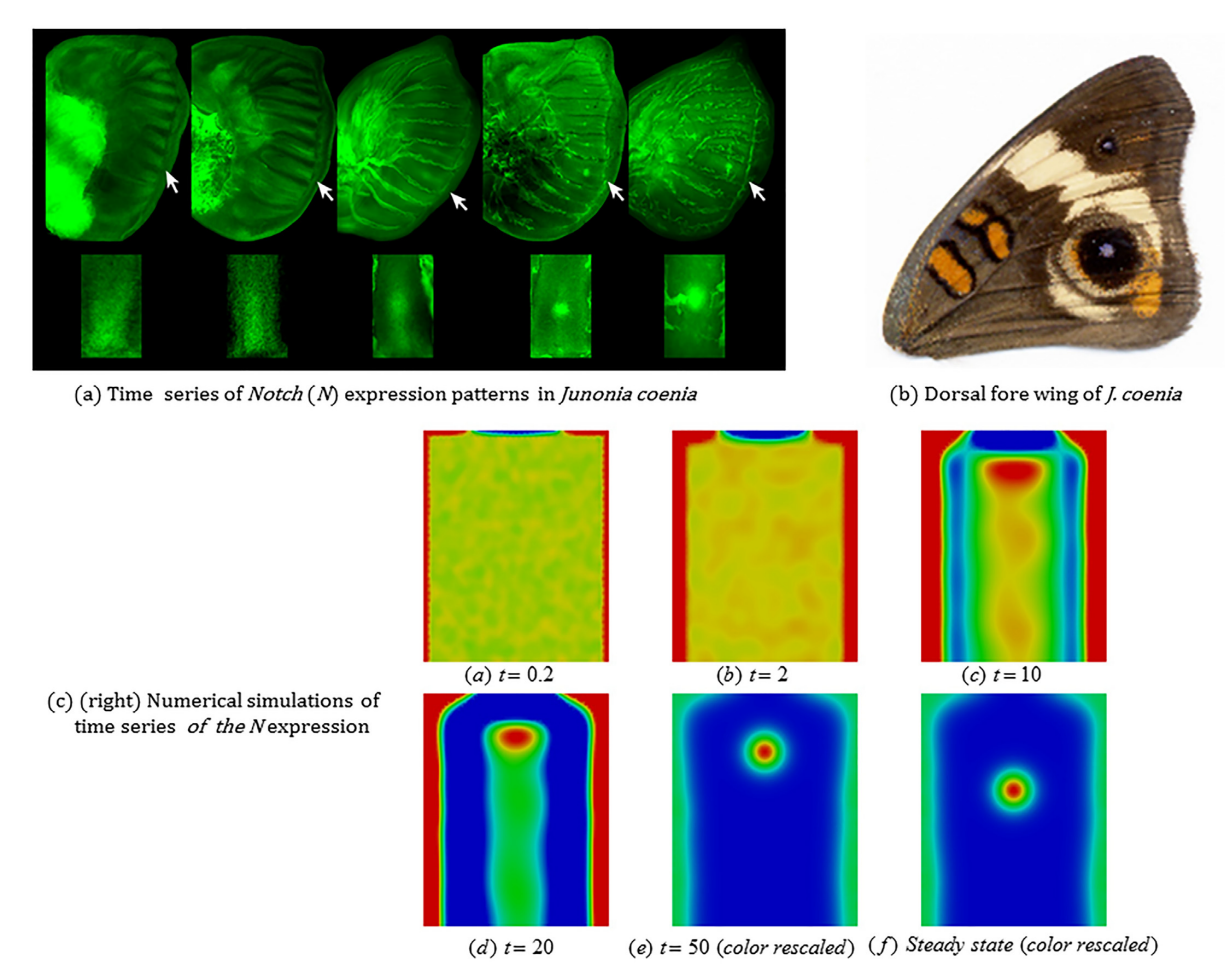
Development of focus points in the wing disc during eyespot determination. (a) Time series of *Notch* expression patterns in *Junonia coenia* wing discs for the final instar eyespot determination. The *Notch* expression patterns were obtained by anti-*N* mouse monoclonal antibody and were visualized on a fluorescent light microscope [18]. (Upper row) The five panels show stained wing discs. (Bottom row) The five panels show the wing cells extracted from the respective figures in the upper panels. Regarding the orientation of bottom panels, the upper side corresponds to the proximal boundary and the bottom side corresponds to the distal boundary of the wing cell, respectively. Insets in the panels detail gene expression in the wing cells marked by white arrows. (b) The corresponding adult forewing of *J*.*coenia*. (c) Simulation results of Fig 7 (a) by use of Eq (3.1). The initial data and boundary conditions are perturbed by uniformly distributed noise which leaves the qualitative features of the results unchanged. In Fig 7 (a), we could see a migration of the focal point into the distal direction from the 3^rd^ stage (middle) to the 4^th^ stage (next to the middle). Both photos (a) and (b): courtesy of Dr. Robert Reed of Cornell University.

Fig 7(A) shows time series of *Notch* (*N*) expression patterns in *Junonia coenia* wing discs during eyespot focus determination and Fig 7(B) is the adult fore wing of *J*. *coenia* [18]. The *N* expression patterns are divided into five stages: (1) broad expression in intervenous regions, (2) upregulation along intervein midlines with no obvious expansion of focal expression, (3) upregulation along intervein midlines with an obvious expansion of focal expression, (4) upregulation in five well-defined foci, with little or no midline expression, and (5) strong upregulation in posterior-most focus, with four anterior foci being greatly reduced or undetectable. From the 3^rd^ stage (middle) to the 4^th^ stage or the 5^th^ stage (right-hand most) in Fig 7(A), we can see a migration of the focal point into the distal direction along the midline of the wing cell. Since the distal margin of the wing cell of the 5^th^ stage could not be seen clearly, the migration might have completed during the time period between the 4^th^ stage and the 5^th^ stage. In any case, the migration of the focal point is reproduced in numerical simulations of our mathematical model as seen in Fig 7(C) (see also Fig 4).

Fig 7(C) shows snapshots of a simulation of the focus point formation shown in Fig 7(A). The domain, that represents a single wing cell, is taken to be a rectangle of length (proximal-distal) 2.5 and width (anterior-posterior) 2. In order to incorporate natural variation in the modeling, we consider boundary conditions on the veins of the form $2a_{1}^{ss}(1+\eta (x))$ and for the proximal boundary we used boundary conditions of the form

$$u(\vec{x})=\left(1-{\text{sin}}^{2}\left(\frac{\pi \vec{x}}{w}\right)\right)2a_{1}^{ss}(1+\eta (x)),$$ (3.5)

where *η* is a uniformly distributed random variable with range [−0.1,0.1]. Similarly the initial data was taken to be the steady state values perturbed by *η*. We see in Fig 7(C) that the results appear insensitive to this moderate level of noise and that the qualitative features are similar to those seen in the simulations and experiments shown in Figs 4 and 7, respectively. A centerline peak forms leaving behind a single focus point which then migrates in the distal direction as is observed in experiments. The incorporation of noise in the boundary conditions appears to destroy the strong symmetry observed in the other simulation as illustrated in the asymmetric nature of the centerline peak.

#### 3.3.2 Abnormal pattern resulting from incomplete vein development

**Fig 8:**
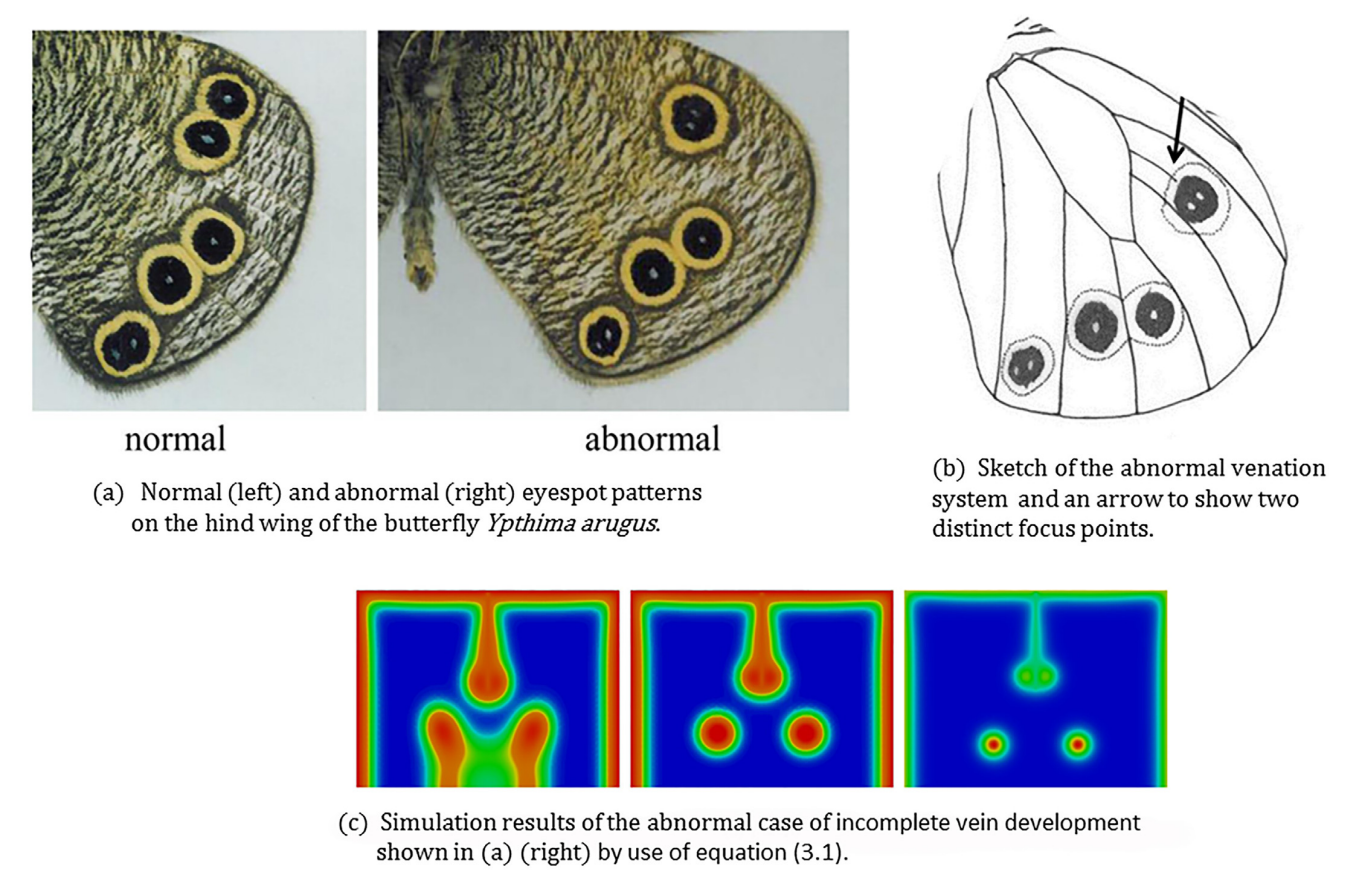
Incomplete vein development leaves two focus points with an eyespot covering two focus points. (a) Normal (left) and abnormal (right) eyespot patterns on the hind wing of the butterfly *Ypthima arugus*. (b) Sketch of the abnormal venation system and an arrow to show two distinct focus points. (c) Simulations of the abnormal case of incomplete vein development shown in (a) (right) by use of Eq (3.1). This incomplete vein development leads to two focus points forming close to both the incompletely developed vein’s end point. The eventual pattern observed on the butterfly wing is that of a single eyespot generated by two focus points that are in close proximity. The corresponding normal pattern is of two distinct eyespots with orally separated foci. Photos (a) and the sketch (b): courtesy of Mr.Toru Tokiwa.

Fig 8 shows an abnormal eyespot pattern of the hind wing of the butterfly *Ypthima arugus* and for comparison the corresponding normal pattern. The left hand subfigure (a) shows two patterns: normal ventral hind wing pattern (left) and the corresponding abnormal pattern in which a vein did not fully develop. To illustrate the scenario during abnormal development of the vein, we include a sketch (right hand subfigure (b) of Fig 8) of the venation system and also an arrow in the picture where we see two distinct focus points and only one eyespot covering the two focus points.

Fig 8(C) illustrates the simulation results on a domain representative of the abnormal case of Fig 8. The domain is a rectangle of width (left to right) four and length three representing two neighboring wing cells. The incompletely developed vein is modeled as an interior boundary originating from the midpoint of the proximal (top boundary) and extending halfway into the interior of the rectangle. For this simulation we selected boundary conditions to be four times the steady state on the completely developed veins and the proximal boundary and twice the steady state on the incompletely developed vein. The interior boundary was modeled as a Dirichlet boundary only for the activator whilst for the inhibitor all the boundaries were taken to be zero-flux. The inclusion of such an interior boundary in the finite element simulations is straightforward once a triangulation is defined over the desired geometry.

We see that with this choice of boundary profiles the resulting focus point distribution is similar to that observed in the case of abnormal wing venation. The results suggest that the incomplete vein may constitute a smaller source of activator than completely developed veins and this could account for the variation in the position of focus points and hence the resultant eyespot pattern.

#### 3.3.3 One eyespot splits into two eyespots through the addition of a vein

**Fig 9:**
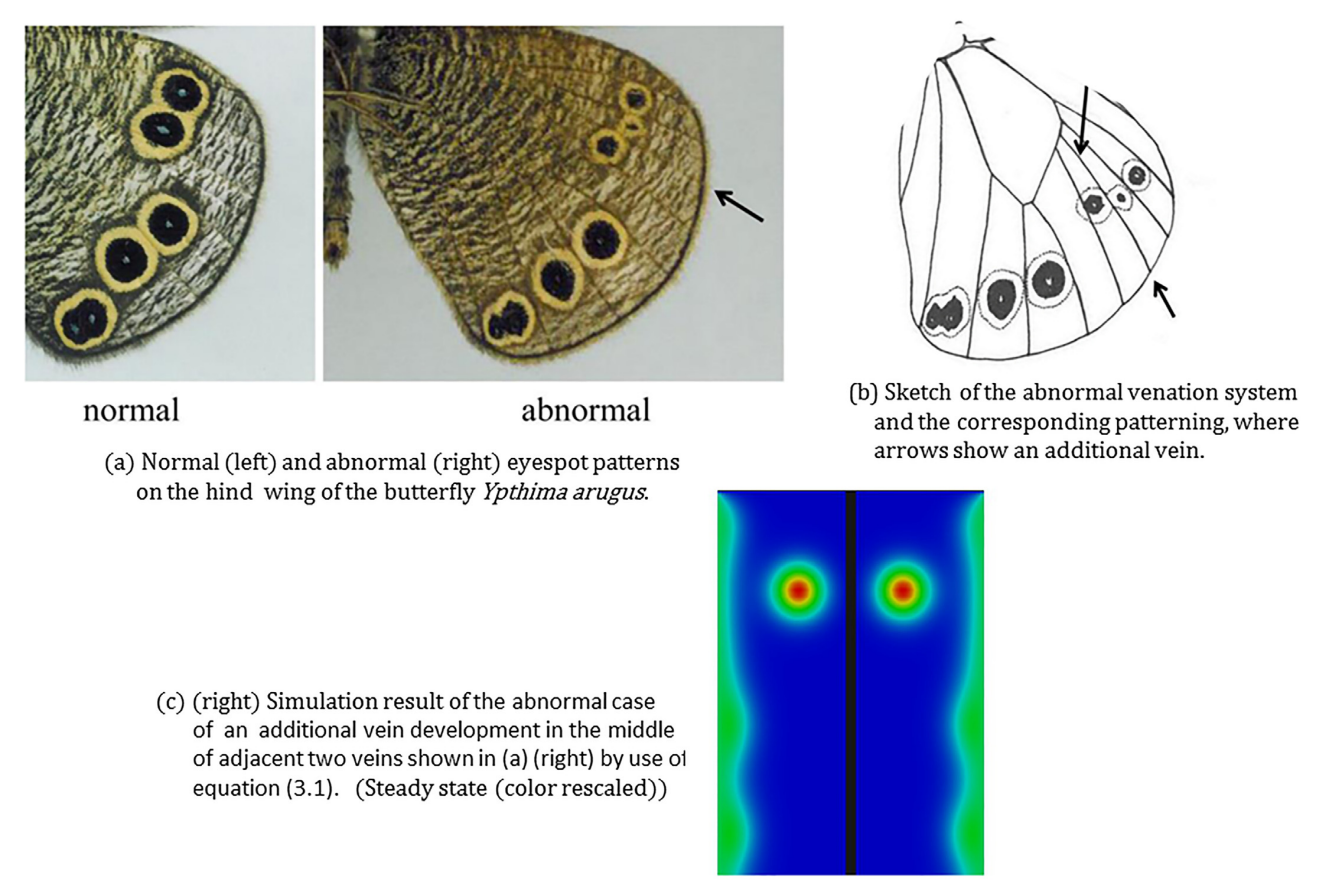
One eyespot splits into two eyespots through the addition of a vein. (a) Normal (left) and abnormal (right) eyespot patterns on the hind wing of the butterfly *Ypthima arugus*. (b) Sketch of the abnormal venation system and the corresponding patterning, where arrows show an additional vein. (c) Simulations of the abnormal case of an additional vein development in the middle of adjacent two veins shown in (a) (right) by use of Eq (3.1). Photos (a) and the sketch (b): courtesy of Mr.Toru Tokiwa.

Finally, we conclude this subsection with another example of an abnormal eyespot pattern on the hind wing of the butterfly *Y*. *arugus*, which at first glance appears incompatible with the current model. The left hand subfigure (a) of Fig 9(A) shows a normal ventral hind wing pattern (left) and the corresponding abnormal pattern, in which an additional vein has developed in the middle of two adjacent veins (right) of the butterfly *Y*. *arugus*. Each of the two newly produced wing cells has one eyespot or focus point, respectively. The width of each wing cell is, of course, narrower than that of the normal width. To illustrate the scenario during abnormal insertion of a vein, we include a sketch (right hand subfigure (b) of Fig 9) of the venation system and also an arrow in the picture where we see the additional foci. The results of Fig 9(C) appear incompatible with our model, as the results in S1 Appendix (i.e., the influence of aspect ratio on focus point determination) show that, in general, reducing the width of the wing cell leads to the formation of fewer foci. However once again if we assume that the abnormal case corresponds to a change in the venation system, specifically, a change in the boundary conditions at the newly formed vein then the model is capable of generating results consistent with the experimental observations. Fig 9(C) shows results of a simulation on two rectangles of length three and width one, i.e., wing cells of half the usual width. The black line in the figure indicates the new vein. We assume that this new vein acts as a homogeneous Dirichlet boundary for the activator. The proximal boundary is also taken to be a homogeneous Dirichlet boundary for the activator whilst the other pre-existing vein is assumed to be a Dirichlet boundary, with the concentration at the boundary at twice the steady state. Due to symmetry, we only solve on a single wing cell and simply reflect along the line of the new vein. We clearly see the formation of two focus points, one in each thin wing cell. Hence under our model one prediction is that for abnormal scenarios such as those in Fig 9 lower activator source strengths on the abnormally inserted veins may account for the abnormal patterning.

## A 2-Stage Model for Focus Point Selection

In Section 3.2, we illustrated that the consideration of different proximal boundary conditions is sufficient to explain focus point selection in a single wing cell. In order to present a complete model for focus point selection, it remains to develop a mechanism for the generation of the proximal boundary profiles. We propose a 2-stage process whereby the first stage consists of the formation of the pattern generating the proximal boundary profiles and the second stage consists of the focus point formation model described in Section 3.1. Although we consider a 2-stage model in this work for simplicity, however, it is certainly of interest mathematically and may be biologically important to consider models where the boundary profile pattern formation process occurs on the same timescale as the focus point formation process. Such a coupled bulk-surface system may be an interesting direction for future research.

### 4.1 A model for the generation of the proximal boundary condition and a 2-stage model for focus point patterning

For the generation of the proximal boundary profiles, we propose an 1-dimensional (1*D*) pattern formation model posed on the proximal boundary Γ_*p*_ = ⋃_*i*_Γ_*p*,*i*_, the union of the proximal boundaries of the wing cells. Clearly, a large variety of models could generate boundary conditions of the form considered in the previous section, the 1*D* model we present here is just one concrete example.

To illustrate the modeling, we work with a concrete example, the activator depleted substrate model of Schnakenberg [19] (see also Murray [20]). The reaction-diffusion system (RDS) is posed on the anterior-posterior margin of the entire wing disc, i.e., the proximal boundary Γ_*p*_ and we assume zero-flux boundary conditions. We consider the following dimensionless RDS for the concentrations of two chemicals (activator and substrate): Find $\vec{u}(x,t)={\left(u_{1}(x,t),u_{2}(x,t)\right)}^{T}$ such that

$$\left\{ \begin{array}{l} \partial_{t}u_{1}(x,t)-d_{1}\Delta_{\Gamma }u_{1}(x,t)=\gamma (x)(k_{1}-u_{1}+u_{1}{}^{2}u_{2})\;on\;\Gamma_{p}\;,\\ \partial_{t}u_{2}(x,t)-d_{2}\Delta_{\Gamma }u_{2}(x,t)=\gamma (x)(k_{2}-u_{1}{}^{2}u_{2})\;on\;\Gamma_{p}\;,\\ \nabla_{\Gamma }u_{1}\cdot \nu =\nabla_{\Gamma }u_{2}\cdot \nu =0\;on\;\partial \Gamma_{p}\;,\\ \vec{u}(x,0)={\vec{u}}_{0}(x)\;on\;\Gamma_{p}\;, \end{array}\right.$$ (4.1)

where, *d*_1_, *d*_2_, *k*_1_ and *k*_2_ are all positive constants. ∇_Γ_ and Δ_Γ_ denote the surface gradient and Laplace-Beltrami operators, respectively. Usually the function *γ* appearing in Eq (4.1) is taken to be a positive constant (which may be interpreted as being related to the domain size or alternatively may be interpreted as a reaction rate [20]). However, in general due to the inherent uniform wavelength associated with Turing patterns, we believe it is not possible to generate boundary profiles such as those considered in Section 3.1, which allow focus points to be generated in arbitrary wing cells with constant parameters if one considers only two component RDSs. We propose here the 1 *D* continuous two component RDSs (4.1) with a spatially varying *γ* to obtain different proximal boundary profiles. We note that 3 or more component RDSs have much richer behavior than 2 component RDSs [21 and 22] and hence may be attractive candidates for the generation of the anterior-posterior pattern.We remark, that this anterior-posterior patterning may occur on a different timescale to the focus point formation process, and it may even occur at an earlier stage of the focus point development, and it would then lay down a pre-pattern for the formation of the proximal boundary profile.

We now describe the 2-stage model we propose for the modelling of focus point patterning on butterfly wings:

Stage 1: In the first stage, the 1*D* RDS (4.1) is solved on the proximal boundary Γ_*p*_ (i.e., the union of the proximal boundaries of the wing cells) to steady state.

Stage 2: In the second stage, independent bulk RDSs of the form (3.1) are solved, i.e., each RDS is posed in a single wing cell and the pattern formation process in this stage occurs independently in each wing cell. The Dirichlet (fixed) boundary conditions on the proximal boundary, $u(\vec{x})$ in Eq (3.1), are taken to be functions of the (patterned) steady state values of the solution to the 1*D* RDS from Stage 1.

In the next section, we present firstly (1) simulation results of Eq (4.1) in cases where the function *γ* is constant, and secondly (2) simulation results of Eq (4.1) with a spatially varying *γ*.

### 4.2 Simulation results of the 2-stage model

We consider the 2-stage model for the selection of focus points described in Section 4.1. The Dirichlet boundary conditions on the proximal boundary given by $u(x)=c_{p,1}{\bar{u}}_{1}(x)+c_{p,2}{\bar{u}}_{2}(x)$, for *x* ∈ Γ_*p*_, where *c*_*p*,1_, *c*_*p*,2_ ∈ *R*. ${\bar{u}}_{1}(x),\;{\bar{u}}_{2}(x)$ are the (patterned) steady state solution values of the 1*D* RDS (4.1).

We reused the parameter values given in Table 1 for the system (3.1) and the remaining parameters were taken as shown below. Thus as the parameter *c*_*p*,2_ = 0, the Dirichlet boundary conditions *u* in Eq (3.1) are simply given by one third of the (patterned) steady state activator concentration, *u*_1_ of the 1*D* RDS (4.1). The initial conditions for the 1*D* RDS are taken as small quasi-random perturbations around the uniform steady state ${\left(\kappa_{1}+\kappa_{2},\kappa_{2}\;/\;{\left(\kappa_{1}+\kappa_{2}\right)}^{2}\right)}^{T}$ and the initial conditions for the bulk RDS are taken as in Section 3.1 (uniform steady state values). In all the simulations, we assume the idealized geometry depicted in Fig 3 (right) consisting of seven rectangular wing cells. We assume the proximal (and marginal boundaries) of each wing cell are of length two and the veins are of length three. Thus the union of the proximal boundaries Γ_*p*_, on which the 1*D* RDS (4.1) is posed is a line of length fourteen (the union of seven proximal wing cell boundaries).

#### 4.2.1 Case study where the function *γ*(*x*) is constant

We start by considering three cases where the function *γ*(*x*) in the 1*D* RDS (4.1) is constant. Firstly we set *γ*(*x*) = 0.01 and *γ*(*x*) = 1 with *κ*_1_ = 0.1, *κ*_2_ = 0.9, *d*_1_ = 0.01, *d*_2_ = 1, *c*_*p*,1_ = 1/3 and *c*_*p*,2_ = 0. For the third case, we set *γ*(*x*) = 5.4 with *κ*_1_ = 0.1, *κ*_2_ = 0.9, *d*_1_ = 1, *d*_2_ = 1, *c*_*p*,1_ = 1.4 and *c*_*p*,2_ = 0.

**Fig 10:**
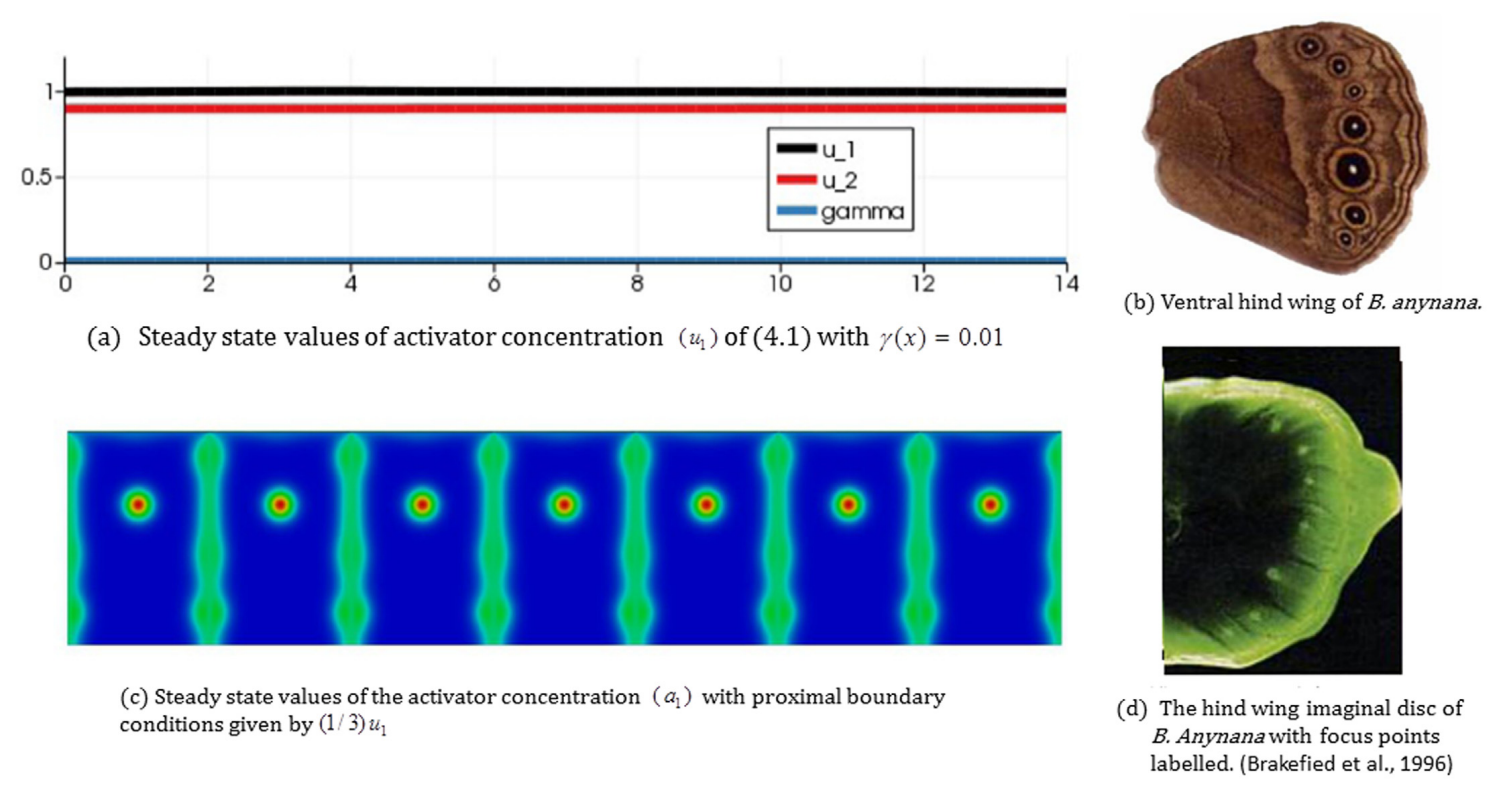
Focus points on the ventral hind wing of *Bycyclus anynana* and numerical simulation results by the 2-stage model. (a) Steady state values of activator concentration (*u*_1_) of the 1*D* RDS (4.1) with *γ*(*x*) = 0.01. (b) Ventral hindwing of *B*. *anynana*. (c) Steady state values of the activator concentration (*a*_1_) for the seven independent bulk RDSs (3.1) with proximal boundary conditions given by (1/3)*u*_1_ where *u*_1_ is the steady state activator concentration shown above. (d) The hind wing imaginal disc of *B*. *anynana* with focus points labelled. (Left hand column) Simulation results of the 2-stage model for focus point formation with a small constant value of the reaction rate *γ* appearing in the 1*D* RDS (4.1) (*γ*(*x*) = 0.01). The model generates a focus point in every wing cell. (Right hand column) The adult ventral hind wing of *B*. *anynana* with seven eyes-pots (top) and the fifth-instar hind wing imaginal disc displaying a pre-pattern with seven foci (bottom), which correspond to eyespots positions on the adult ventral hind wing. Experimental figures: from Brakefield et al. [2] with permission by the publisher.

Case 1: Seven focus points on the ventral hindwing of *Bycyclus anynana*: Brakefield et al. [2] examined *Dll* expression in the late fifth-instar *Bicyclus anynana* ventral hind wing imaginal disc. They found that it is in a broad distal band and at high levels in seven focus points, which correspond exactly to the future positions of the seven eyespots on the adult ventral hind wing. In Fig 10, the top right photo (b) shows the adult ventral hind wing of *B*. *anynana* with seven eyespots, and the bottom right (d) is the fifth-instar hind wing imaginal disc displaying a pre-pattern with seven focus points of *Dll* expression.

On the other hand, (left hand column): (a) and (c) of Fig 10 show results of the 2-stage model with *γ*(*x*) = 0.01. In this case, *γ* is below the critical value for the onset of diffusion driven-instability globally and the solution to the 1*D* RDS (4.1) simply converges to the uniform steady state. Hence, we have a constant value for the proximal boundary condition. As the choice of the coupling coefficient *c*_*p*,1_ is such that the proximal boundary condition $\left(c_{p,1}{\bar{u}}_{1}\right)$ is below the critical value for focus point formation, we generate a focus point in every wing cell. The resultant pattern is similar to that observed in the developing wing disc of *B*. *anynana* as shown in Fig 10 (right hand column).

**Fig 11:**
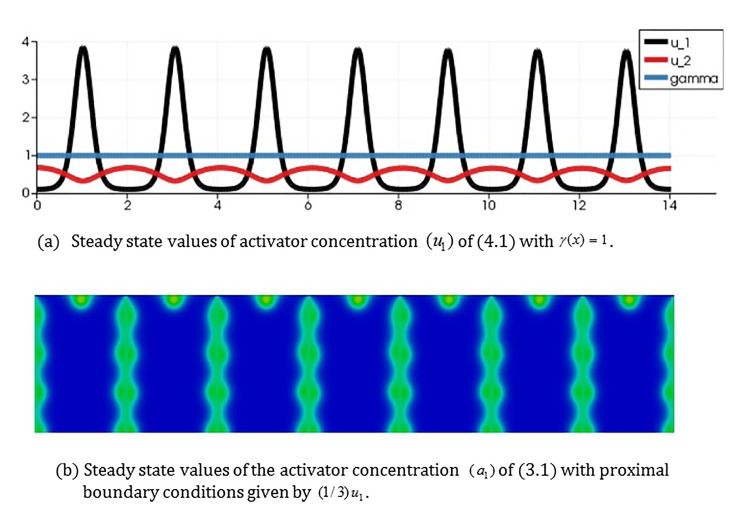
Simulation results of the 2-stage model for focus point formation with a large constant value of the reaction rate *γ* appearing in the 1*D* RDS (4.1) (*γ*(*x*) = 1). The model generates no focus points. (a) Steady state values of activator concentration (*u*_1_) of the 1*D* RDS (4.1) with *γ*(*x*) = 1. (b) Steady state values of the activator concentration (*a*_1_) for the seven independent bulk RDSs (3.1) with proximal boundary conditions given by (1/3)*u*_1_ where *u*_1_ is the steady state activator concentration shown above.

Case 2: No focus points on the wing: Fig 11 shows results of the coupled model with *γ*(*x*) = 1. In this case, *γ* is above the critical value for the onset of diffusion driven instability and we obtained a patterned steady state solution to the 1*D* RDS (4.1). The solution profile is one of seven equally spaced activator peaks in the domain with the activator profile on each proximal boundary ([0,2], [2,4],…) appearing similar in shape to the convex profile of Fig 5. When we simulate the 2-stage model with this boundary profile and the selected coupling coefficient *c*_*p*,1_, we observe similar behavior to the simulations of Fig 5 (with the convex profile) with no focus points forming in any of the wing cell.

Beldade et al. [4] did artificial selection to examine how the relative size of the anterior and posterior eyespots on the dorsal forewing of *B*. *anynana* can be changed in a laboratory population. They got almost all possible phenotypes by generations G25, e.g., females with no eyespots, with only one anterior eyespot, only one posterior eyespot, two (anterior and posterior) eyespots, and extra, satellite eyespots on the entire dorsal forewing. Simulation results of Fig 11(B) correspond to *B*. *anynana* females having no eyespots.

**Fig 12:**
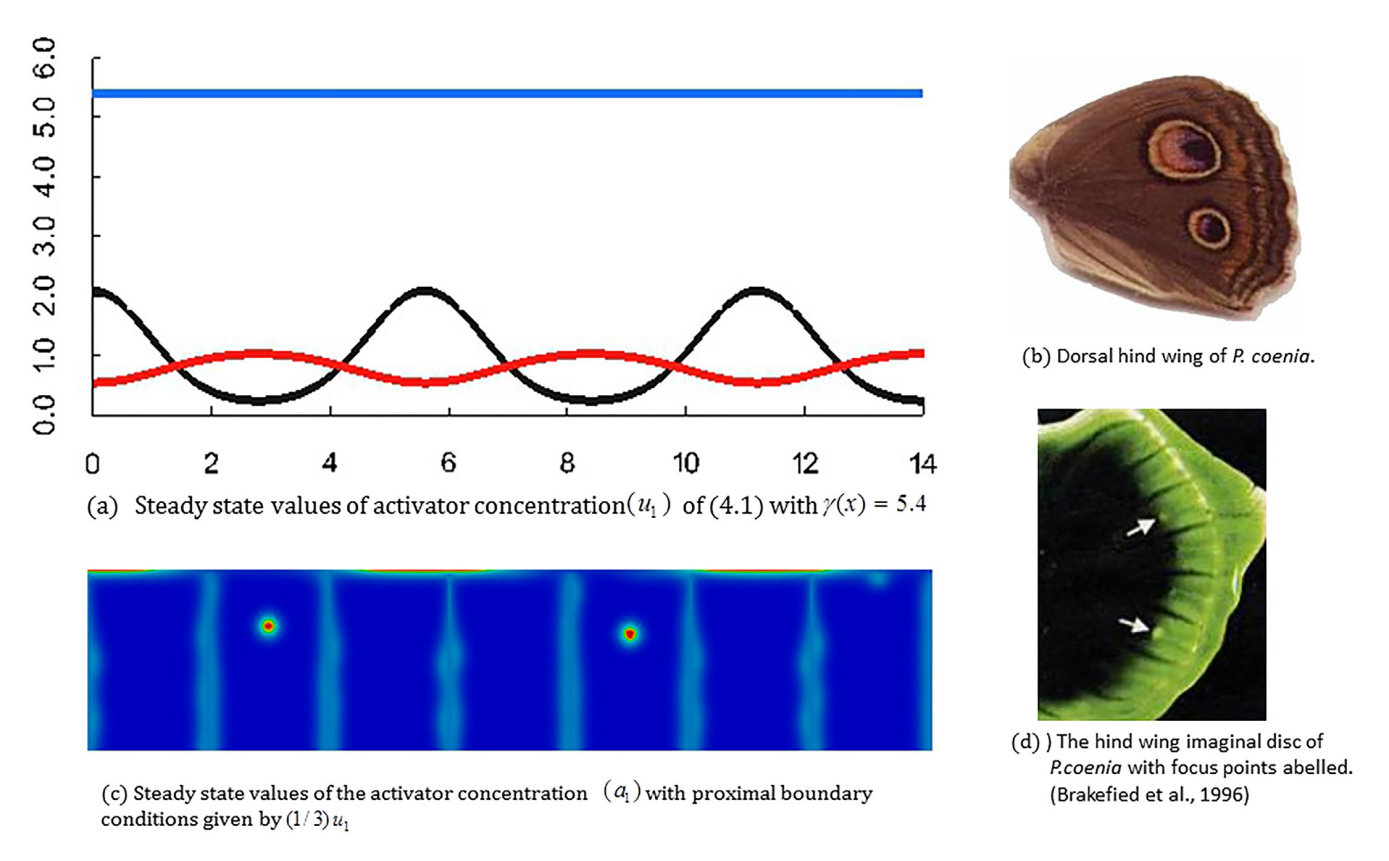
Focus points on the dorsal hind wing of *Precis coenia* and numerical simulation results by the 2-stage model. (a) Steady state values of activator concentration (*u*_1_) of the 1*D* RDS (4.1) with a constant value of the function *γ*(*x*). (b) Dosal hindwing of *P*. *coenia*. (c) Steady state values of the activator concentration (*a*_1_) for the seven independent bulk RDSs (3.1) with proximal boundary conditions given by (1/3)*u*_1_ where *u*_1_ is the steady state activator concentration shown above. (d) The hind wing imaginal disc of *P*.*coenia* with focus points labelled. Two white arrows point two *Dll* stained focus points. (Left hand column) Simulation results of the 2-stage model for focus point formation with aconstant value of the reaction rate *γ* appearing in Eq (4.1) (*γ*(*x*) = 5.4). The model generates the formation of foci in wing cells 2 and 5 and no foci in the other wing cells similar to the experimental observations. (Right hand column) The adult *P*. *coenia* dorsal hindwing with two eyespots (top) and the fifth-instar hindwing imaginal disc displaying a pre-pattern with two foci (bottom), which correspond to eyespots positions on the adult dorsal hindwing. Experimental figures: from Brakefield et al. [2] with permission by the publisher.

Case 3: Two focus points on the dorsal hindwing of *Precis coenia*: Brakefield et al. [2] showed that the fifth-instar *Precis coenia* hindwing imaginal disc exhibits two spots of *Dll* expression, which correspond to the future positions of two eyespots on the adult hindwing. In Fig 12, the top right photo (b) shows the adult *P*.*coenia* dorsal hindwing with two eyespot focus points, and the bottom right (d) is the fifth instar *P*. *coenia* hindwing imaginal disc displaying a pre-pattern with two focus points of *Dll* expression, which correspond to the position of the eyespots on the adult dorsal hindwing.

By selecting, the parameters in the two component RDS posed on the proximal boundary corresponding to the third case described above, we generate patterns with a larger wavelength (due to the increased diffusion coefficients). As shown in Fig 12(A), the resultant steady state consists of an activator pattern with only two interior minima in the domain corresponding to the proximal boundaries of wing cells two and five. When we simulate the 2-stage model with this boundary profile and the selected coupling coefficient *c*_*p*,1_, we observe the formation of foci in wing cells 2 and 5 and no foci in the other wing cells similar to the experimental observations of *P*. *coenia* as shown in Fig 12 (right hand column).

#### 4.2.2 Simulation results of (4.1) with a spatially varying *γ* in the anterior-posterior direction

As mentioned in Section 4.1, through the consideration of 2-component RDSs with constant parameters, for the 1*D* patterning mechanism appears insufficient to generate boundary profiles leading to focus points in an arbitrary wing cells. A major difficulty lies in the fact that Turing patterns typically possess a constant wavelength over the domain, hence patterned profiles in one region of the domain with no patterning (convergence to the homogeneous steady state) in another region is not possible. However, such a pattern distribution is easily achieved through the consideration of systems with spatially varying parameters. See for example [23] for previous work in this direction.

In upper figures in S2 Appendix, we present simulation results of Eq (4.1) (as part of the coupled model) that illustrate that a system of the form (4.1) with a spatially varying *γ*, specifically an anterior-posterior gradient in *γ* can restrict patterning to certain portions of the wing (see also the corresponding bottom figures in S2 Appendix). RDSs with spatially varying parameters have been the subject of much study in the literature, for example [24]. We remark that numerical studies suggest patterning can be restricted to certain portions of the domain through the use of spatially varying parameters similar to the results we report on the current work. In [25, 26], the authors model the regulation of digit patterning of developing vertebrate limb buds by *Hox* genes using a Turing RDS. Their results indicate that changing the kinetic parameters can influence the wavelength of the resultant pattern. They also consider spatial gradients in kinetic parameters, and show that this allows the robust formation of a striped pattern with a given orientation that models digit formation.

## Summary and Discussion

In this study, we presented a model for the selection and distribution of eyespot focus points on the wings of Lepidoptera. The basic idea of the model is that the wing cells, in which eyespot foci are formed, are selected by the source value of an activator on the proximal veins of the entire wing disc. Specifically a variable proximal boundary condition in the anterior-posterior direction of the entire wing disc governs focus point selection. Through numerical simulations on idealized wing disc geometries, we illustrated that this proximal wave-like boundary condition can govern the number and locations of eyespot focus points on the wing surface. As a result, the model could provide a plausible mechanism for the selection of global eyespot focus points on wing discs of some butterfly species such as *Junonia* (or *Precis*) *coenia*, *Bicyclus anynana*, and *Ypthima arugus* in the Nympalidae family.

Our study suggests that a key factor that determines focus point selection could be in the overall venation system of the wing disc. We assumed that the veins are sources of the activator [9], i.e., they act as Dirichlet boundaries in the mathematical model and numerical simulations. We first considered a number of different prescribed boundary conditions on the proximal boundary, and our results show that one may construct boundary profiles (which could be smooth and continuous or discontinuous across the whole wing disc) such that focus points may be selected in any wing cell and hence may reproduce the variety of focus point distributions observed in experiments. To complete the model, we then proposed a simple 1*D* reaction-diffusion model for the generation of the proximal boundary condition profile, that is, a surface Turing system posed in the anterior-posterior direction on the proximal vein.

We stress that the key factor is a change in source values of the activator on the proximal veins in the anterior-posterior direction of the wing disc and this change may be realized by a variety of different patterning mechanisms. One of our main results is that under our model, wing cells in which eyespot focus points are generated need to have lower source strength of the activator on the proximal boundary than wing cells that do not produce focus points. We also stress that the number and locations, that is, the global distribution of eyespot foci on the wing disc could be largely controlled by two gradients along two different directions, that is, the first one is the gradient in spatially varying parameters such as the reaction rate *γ* in Eq (4.1) along the anterior-posterior direction on the proximal boundary of the wing cells (see Section 4.2.2 and S2 Appendix), and the second one is the gradient in source values of the activator along the veins in the proximal-distal direction of the wing cell. The first gradient could determine the number and locations of foci on the wing cells. The second gradient could control the location of the focus point in the proximal-distal direction within the wing cell (see also discussion in Section 3.2).

The role of boundary conditions in the determination of patterning generated by reaction-diffusion systems has been the subject of previous work. For example, Dillon et al. [27] show that changes in boundary conditions can have a profound influence on the solutions both in terms of existence and uniqueness and in terms of the stability of patterns. Page et al. [24] investigate Turing systems with a discontinuity in a kinetic parameter and show that such a system may be decomposed into systems with constant parameters and anomalous boundary conditions. They further show that such systems may exhibit spatial patterns outside the classical Turing space. Barrio et al., [28] and Aragon et al., [29] consider the role of boundary conditions in Turing reaction-diffusion system models for pigment patterns on the skin of fish. In particular, they observe that the consideration of different boundary conditions may increase the number of scenarios such models are capable of explaining. In light of our numerical results and the theoretical and numerical works mentioned above, further mathematical investigations in the same direction as the works above into the role of boundary conditions in patterning by Turing systems are certainly warranted.

The current model framework consists of wing discs that are the union of several (identical) rectangular wing cells, although we also considered a somewhat more representative wing cell with curved (proximal and wing margin) boundaries and varying width. This modeling framework might be improved by considering a more realistic geometry of the wing cells, rather than the rectangular cells considered in this study, although experimental results suggest that during the time at which focus points form, a rectangular cell is a good approximation. Preliminary numerical results suggest that other forms of boundary conditions can generate more complicated foci such as arc shaped foci or multiple foci of different sizes in a wing cell. Such models could perhaps reproduce some of the diversity of focal shapes that are occasionally observed in nature. The exploration of such possibilities are reserved for future work. Here our objective has been to provide a proof of concept that anterior-posterior patterning alone may determine focus point selection.

## Supporting Information

S1 Appendix The influence of aspect ratio on eyespot focus point determination. The figures in S1 Appendix show snapshots of the activator concentration corresponding to the solution of Eq (3.1). The wing cells are taken to be rectangular and of fixed length equal to three, but the width is now varied with the width taken to be 1, 1.5, 2, 2.5 and 3 reading from left to right in each subfigure. In the left hand column, the boundary conditions for the activator on the proximal boundary (top) of each rectangular cell are taken to be zero, and in the right hand column, they are set to twice the steady state value. Initially in all but the thinnest wing cell (left hand most in each column), a vertical stripe of high activator concentration is generated originating from the zero-flux distal boundary (bottom). As the width of the wing cells is increased, the midline peak starts to generate multiple spots as it recedes with insertion of new spots in the proximal-wing margin, and in the anterior-posterior direction, both exhibited. There appears to be a monotonic relationship between aspect ratio and number of focus points with wider wing cells (with a fixed length) exhibiting more focus points at steady state.
(TIF)

S2 Appendix Restricting focus point formation to regions of the wing through the use of spatially varying parameters in the anterior-posterior 1*D* RDS. As mentioned in Section 4.2.2, 2-component RDSs with constant parameters alone for the 1*D* patterning mechanism appear insufficient to generate boundary profiles leading to focus points in arbitrary wing cells. However, such a pattern distribution is achieved through the consideration of systems with spatially varying parameters such as the reaction rate *γ*(*x*) in Eq (4.1). To illustrate this effect, in figures in S2 Appendix, we report on the steady states for the 1*D* systems obtained using a monotonically increasing gradient for the reaction rate *γ*(*x*) = (*x*/14)^*p*^, with *p* = 1, 2 and 8 ((a), (b), and (c), respectively). The remaining parameters were taken to be with *κ*_1_ = 0.1, *κ*_2_ = 0.9, *d*_1_ = 0.01, *d*_2_ = 1, *c*_*p*,1_ = 1/3 and *c*_*p*,2_ = 0. As the gradient of this function is decreased (smaller *p*), focus points form only closer to the anterior margin whilst for larger gradients focus points can be made to form on almost the entire wing. The case *p* = 0, corresponds to Fig 11 with no focus points forming, whilst formally in the limit *p*→∞, a focus point forms in each wing cell, as in Fig 10, as the 1*D* RDS solution would be close to the steady state value due to the initial conditions.
(TIF)
