# Supplementary figures and images for: A Model for Selection of Eyespots on Butterfly Wings
Source: PLoS One. 2015 Nov 4;10(11):e0141434. doi: 10.1371/journal.pone.0141434 (PMC4633216; doi:10.1371/journal.pone.0141434)

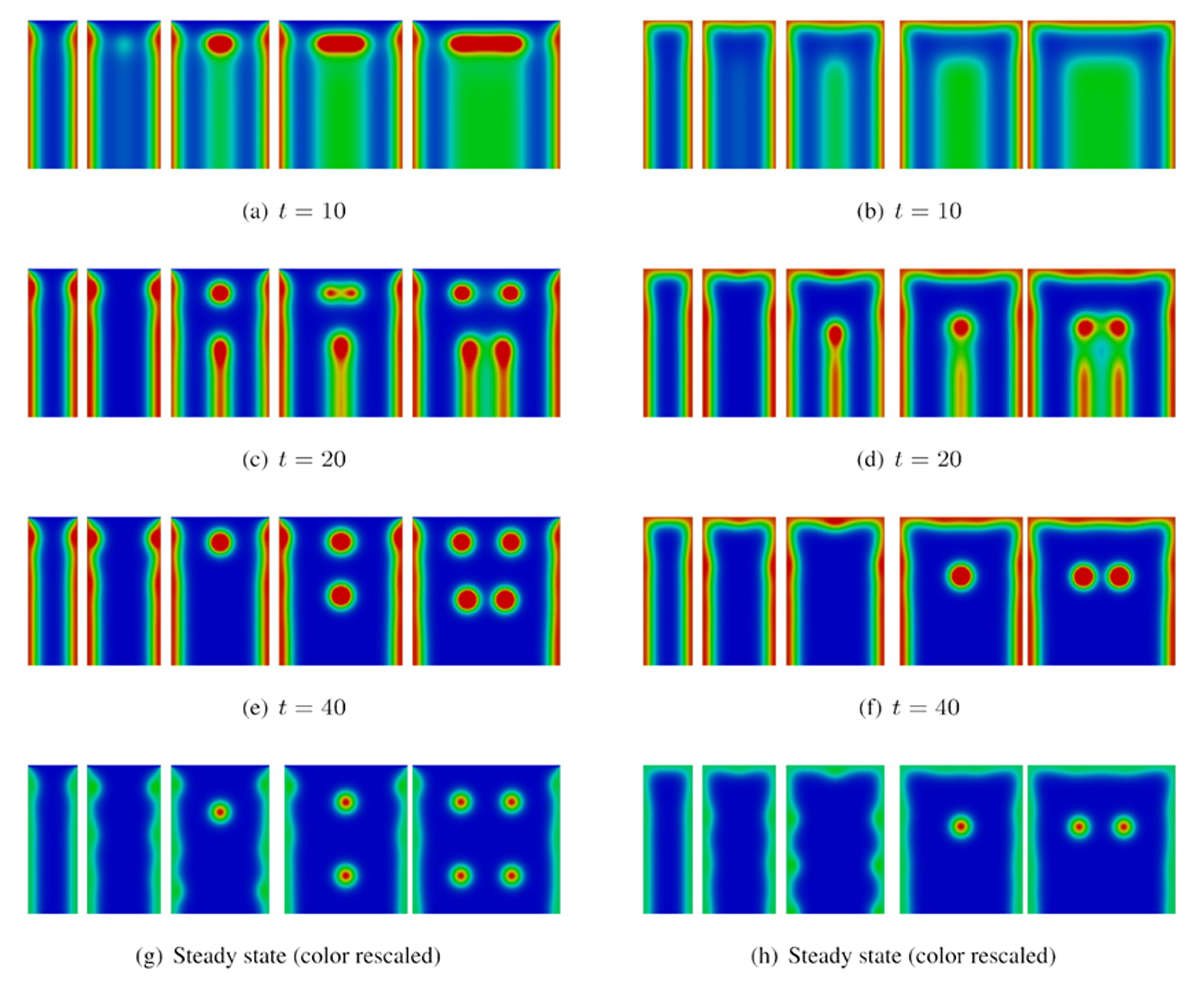

Supplement: S1 Appendix — The figures in S1 Appendix show snapshots of the activator concentration corresponding to the solution of Eq (3.1). The wing cells are taken to be rectangular and of fixed length equal to three, but the width is now varied with the width taken to be 1, 1.5, 2, 2.5 and 3 reading from left to right in each subfigure. In the left hand column, the boundary conditions for the activator on the proximal boundary (top) of each rectangular cell are taken to be zero, and in the right hand column, they are set to twice the steady state value. Initially in all but the thinnest wing cell (left hand most in each column), a vertical stripe of high activator concentration is generated originating from the zero-flux distal boundary (bottom). As the width of the wing cells is increased, the midline peak starts to generate multiple spots as it recedes with insertion of new spots in the proximal-wing margin, and in the anterior-posterior direction, both exhibited. There appears to be a monotonic relationship between aspect ratio and number of focus points with wider wing cells (with a fixed length) exhibiting more focus points at steady state. (TIF) [file pone.0141434.s001.tif]

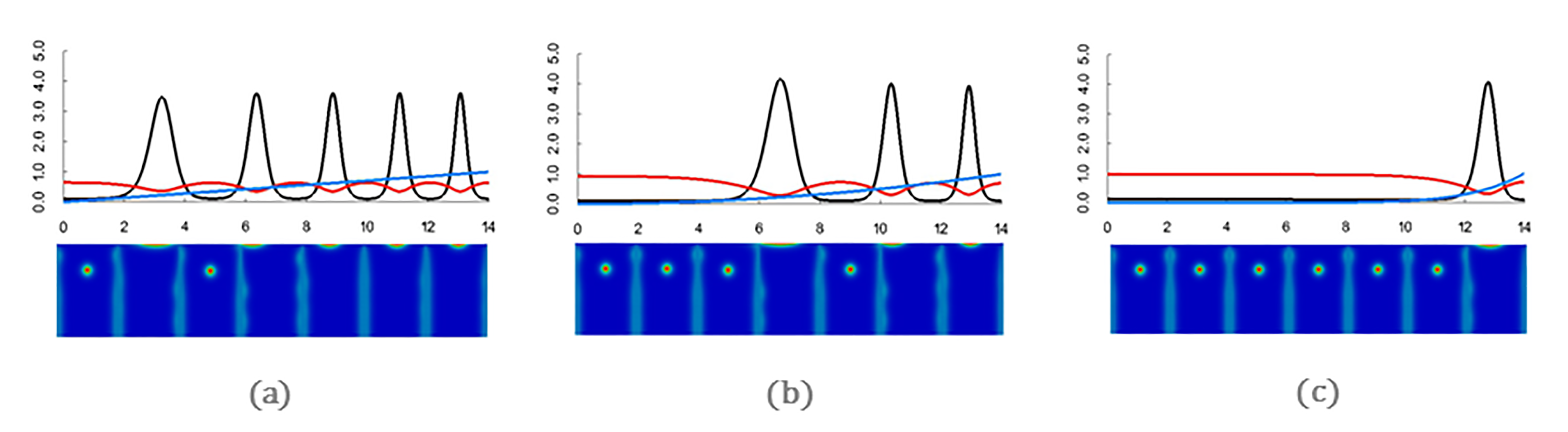

Supplement: S2 Appendix — As mentioned in Section 4.2.2, 2-component RDSs with constant parameters alone for the 1D patterning mechanism appear insufficient to generate boundary profiles leading to focus points in arbitrary wing cells. However, such a pattern distribution is achieved through the consideration of systems with spatially varying parameters such as the reaction rate γ(x) in Eq (4.1). To illustrate this effect, in figures in S2 Appendix, we report on the steady states for the 1D systems obtained using a monotonically increasing gradient for the reaction rate γ(x) = (x/14)p, with p = 1, 2 and 8 ((a), (b), and (c), respectively). The remaining parameters were taken to be with κ 1 = 0.1, κ 2 = 0.9, d 1 = 0.01, d 2 = 1, c p,1 = 1/3 and c p,2 = 0. As the gradient of this function is decreased (smaller p), focus points form only closer to the anterior margin whilst for larger gradients focus points can be made to form on almost the entire wing. The case p = 0, corresponds to Fig 11 with no focus points forming, whilst formally in the limit p→∞, a focus point forms in each wing cell, as in Fig 10, as the 1D RDS solution would be close to the steady state value due to the initial conditions. (TIF) [file pone.0141434.s002.tif]
